# Supplementary material for: Association between Precarious Employment and Chronic Stress: Effect of Gender, Stress Measurement and Precariousness Dimensions—A Cross-Sectional Study
Source: Int J Environ Res Public Health. 2022 Jul 26;19(15):9099. doi: 10.3390/ijerph19159099 (PMC9330896; doi:10.3390/ijerph19159099)
Supplement: Supplementary file 1 [file ijerph-19-09099-s001.zip › ijerph-1813373-supplementary.pdf]

International Journal of
Environmental Research and Public Health

MDPI

Table S1. Goodness-of-ft of EPRES scale from exploratory factor analysis. Precarious Employment and Stress Study sample, 2020, N men = 125, N women = 130.

	Factor 1	Factor 2	Factor 3	Factor 4	Factor 5	Factor 6	Factor 7	Uniqueness
Type of contract			0.26	0.62	−0.16		0.23	0.47
How long in total have you been working for this company?			0.30	0.34	−0.15			0.75
Approximately how much do you earn net per month?	0.21		0.66	0.11	−0.16			0.49
How often does your current salary allow you to cover your basic daily needs?	0.10		0.73					0.43
How often does your current salary allow you to cover major unforeseen expenses?		0.16	0.76	0.11				0.38
How were the working conditions in relation to your working hours decided?		0.11				0.69		0.51
How were the working conditions in relation to your salary decided?						0.67		0.54
How often in this company are you afraid to demand better working conditions?	0.20	0.48						0.72
How often in this company do you feel defenceless against unfair treatment by your superiors?	0.24	0.67				0.12		0.47
How often in this company would you be fired from your job if you did not do everything they asked you to do?		0.34	0.15				0.28	0.77
How often in this company are you treated in an authoritarian way?	0.21	0.70						0.45
How often in this company do they make you feel that you can easily be replaced?	0.11	0.69	0.18					0.48
Entitlement to paternity/paternity leave	0.10		0.22	0.48			−0.28	0.62
Entitlement to retirement pension, disability	0.21		0.40	0.34			−0.26	0.61
Entitlement to unemployment benefit				0.46	0.11	0.13	0.13	0.74
Do you have the right to severance pay?				0.61			−0.13	0.61
In this company you can take your weekly holidays without any problems	0.20					0.19	0.22	0.86
In this company, you can take your holidays without any problems	0.31	0.15	0.15	0.14			0.35	0.71
In this company you can take a day off for family reasons (care of children, dependants, sick people, etc.) without any problem	0.61	0.36		0.19			−0.10	0.44
In this company you can take a day off for personal reasons without any problem	0.68	0.31					−0.13	0.41
In this company you can take sick leave without any problem	0.63		0.18	0.15		0.11	0.19	0.49
In this company, you can go to the doctor when you need to	0.71	0.12	0.11					0.46
Voluntarily work overtime		0.10			0.75			0.41
Number of overtime hours you work					0.76			0.40

Changes in working hours, If yes, how far in advance are you informed of such changes?	0.15	0.14		0.23	−0.18	0.86

Table S2. Goodness-of-ft of EPRES scale from confirmatory factor analysis. Precarious Employment and Stress Study sample, 2020, N men = 125, N women = 130.

	χ2 [df]. *p*-Value	CFI	ΔCFI	TLI	ΔTLI	RMSEA (90% CI)	ΔRMSEA
Regular CFA							
	322.909 [231]; $p < 0.001$	0.989	-	0.987	-	0.040 (0.030–0.050)	-
Multi-group CFA by women and men							
Configural invariance	628.514 [462]; $p < 0.001$	0.982	0	0.979	0	0.054 (0.043–0.064)	0.014
Metric invariance	659.604 [479]; $p < 0.001$	0.981	−0.001	0.978	−0.001	0.055 (0.044–0.065)	0.001
Thresholds invariance	683.268 [541]; $p < 0.001$	0.985	0.004	0.985	0.007	0.046 (0.034–0.056)	−0.009

Table S3. Reliability of EPRES scale from Cronbach´s alpha. Precarious Employment and Stress Study sample. 2020. N men = 125. N women = 130.

Item	Obs	Sign	Item-Test Correlation	Item-Rest Correlation	Average Interitem Covariance	Alpha
Salary						
Approximately how much do you earn net per month?	254	+	0.76	0.59	1.16	0.77
How often does your current salary allow you to cover your basic daily needs?	254	+	0.86	0.67	0.72	0.65
How often does your current salary allow you to cover major unforeseen expenses?	254	+	0.90	0.70	0.54	0.66
Test scale					0.80	0.78
Vulnerability						
How often in this company are you afraid to demand better working conditions?	255	+	0.67	0.44	0.74	0.71
How often in this company do you feel defenceless against unfair treatment by your superiors?	255	+	0.74	0.57	0.68	0.66
How often in this company would you be fired from your job if you did not do everything they asked you to do?	255	+	0.62	0.33	0.81	0.76
How often in this company are you treated in an authoritarian way?	255	+	0.75	0.61	0.70	0.65
How often in this company do they make you feel that you can easily be replaced?	255	+	0.76	0.59	0.64	0.65
Test scale					0.71	0.73
Rights						
Entitlement to paternity/paternity leave.	255+		0.71	0.43	0.07	0.51
Entitlement to retirement pension, disability	255+		0.67	0.37	0.08	0.56
Entitlement to unemployment benefit	255+		0.57	0.29	0.10	0.61
Do you have the right to severance pay?	255+		0.75	0.48	0.06	0.46
Test scale					0.08	0.61
Vulnerability						
In this company you can take your weekly holidays without any problems	255	+	0.44	0.28	0.67	0.79

In this company, you can take your holidays without any problems	255	+	0.59	0.40	0.58	0.76
In this company you can take a day off for family reasons (care of children, dependants, sick people, etc.) without any problem	255	+	0.76	0.60	0.47	0.71
In this company you can take a day off for personal reasons without any problem	255	+	0.79	0.64	0.45	0.70
In this company you can take sick leave without any problem	255	+	0.72	0.56	0.50	0.73
In this company, you can go to the doctor when you need to	255	+	0.74	0.60	0.50	0.72
Test scale					0.53	0.77
Salary						
Voluntarily work overtime	253	+	0.77	0.46	0.19	0.19
Number of overtime hours you work	253	+	0.81	0.49	0.08	0.09
Changes in working hours, If yes, how far in advance are you informed of such changes?	253	+	0.56	0.08	1.08	0.78
Test scale					0.45	0.51

Table S4. Linear regression coefficients and 95% confidence intervals (CI) for EPRES global scale predicting Production of Adrenal and Gonadal steroids, adjusted for age and stratified by sex. Precarious Employment and Stress Study sample, 2020.

**Outomes** [1]	**Men (n = 125)**		**Women (n = 130)**	
	**β**	**95%CI**	**β**	**95%CI**
Adrenal and gonadal steroids				
Cortisol	−0.01	(−0.27–0.26)	−0.01	(−0.23–0.22)
20αDHF	0.10	(−0.24–0.43)	0.19	(−0.08–0.46)
20βDHF	0.05	(−0.14–0.25)	0.13	(−0.03–0.28)
20αDHE	0.06	(−0.14–0.25)	0.24 *	(0.06–0.43)
20βDHE	0.05	(−0.14–0.24)	0.21 **	(0.05–0.37)
Cortisone	0.04	(−0.14–0.22)	0.16	(−0.01–0.33)
Cortolone	0.02	(−0.15–0.20)	0.05	(−0.12–0.21)
Dehidrocortisone	0.11	(−0.06–0.28)	0.17 *	(0.00–0.34)
Testosterone	0.14	(−0.07–0.36)	0.08	(−0.28–0.44)
Androstenedione (AED)	0.08	(−0.09–0.25)	0.15	(−0.05–0.35)
Progesterone	0.12	(−0.54–0.78)	0.05	(−0.49–0.60)
20αDHF/20βDHF	0.03	(−0.21–0.26)	0.06	(−0.11–0.23)
20αDHE/20βDHE	0.01	(−0.07–0.08)	0.03	(−0.04–0.11)
Cotisone _Dehydrocorticosterone (E_A)	−0.07	(−0.24–0.11)	−0.01	(−0.22–0.19)
Cortisol_Cortisona	−0.04	(−0.24–0.16)	−0.16 *	(−0.32–−0.00)
%Cortisol	−0.04	(−0.18–0.10)	−0.16 **	(−0.27–−0.05)
%Cortisone	0.00	(−0.08–0.08)	0.00	(−0.06–0.07)
%20αDHF	0.06	(−0.17–0.30)	0.04	(−0.14–0.21)
%20βDHF	0.02	(−0.08–0.12)	−0.02	(−0.11–0.06)
%20αDHE	0.02	(−0.07–0.11)	0.09 *	(0.00–0.17)
%20βDHE	0.02	(−0.07–0.10)	0.06	(−0.01–0.12)
20αDHF/Cortisol	0.10	(−0.15–0.36)	0.20 *	(0.01–0.38)
20βDHF/Cortisol	0.06	(−0.13–0.25)	0.14	(−0.01–0.28)

[1] All outcomes have been transformed into logarithms to correct skewness. ** $p < 0.01$, * $p < 0.05$.
